# Assessment of the reliability of reproducing two-dimensional resistivity models using an image processing technique

**DOI:** 10.1186/2193-1801-3-214

**Published:** 2014-05-01

**Authors:** Kehinde S Ishola, Mohd NM Nawawi, Khiruddin Abdullah, Ali Idriss Aboubakar Sabri, Kola Abdulnafiu Adiat

**Affiliations:** Geophysics Section, School of Physics, Universiti Sains Malaysia, 11800 Penang, Malaysia

**Keywords:** Image processing, Algorithm librarian, Electrode arrays, Image registration, Model parameters, Errors estimation

## Abstract

This study attempts to combine the results of geophysical images obtained from three commonly used electrode configurations using an image processing technique in order to assess their capabilities to reproduce two-dimensional (2-D) resistivity models. All the inverse resistivity models were processed using the PCI Geomatica software package commonly used for remote sensing data sets. Preprocessing of the 2-D inverse models was carried out to facilitate further processing and statistical analyses. Four Raster layers were created, three of these layers were used for the input images and the fourth layer was used as the output of the combined images. The data sets were merged using basic statistical approach. Interpreted results show that all images resolved and reconstructed the essential features of the models. An assessment of the accuracy of the images for the four geologic models was performed using four criteria: the mean absolute error and mean percentage absolute error, resistivity values of the reconstructed blocks and their displacements from the true models. Generally, the blocks of the images of maximum approach give the least estimated errors. Also, the displacement of the reconstructed blocks from the true blocks is the least and the reconstructed resistivities of the blocks are closer to the true blocks than any other combined used. Thus, it is corroborated that when inverse resistivity models are combined, most reliable and detailed information about the geologic models is obtained than using individual data sets.

## Introduction

Geophysical imaging is used to picture Earth’s subsurface. The use of near surface geophysical methods allows subsurface features to be located, mapped and characterized in response to changes in physical, electrical or chemical properties in the subsurface. The location and orientation of anomalies are essential for modeling of the subsurface geology. In applied geophysics, modeling has become an essential tool for the comparison of the resolution of different direct current resistivity electrode arrays (Martorana et al. 2009). Images obtained are used to map the extent of occurrence of some natural resources in the subsurface and to interpret them on the basis of their physical properties (e.g., electrical conductivity, density).

In order to obtain a high resolution and reliable image, the electrode array used should provide adequate information about the model (Dahlin and Zhou 2004). The selection of the most appropriate electrode arrays for direct current resistivity field surveys has continued to draw attention among researchers in view of the merits and limitations of these arrays. The type of arrays chosen and the model parameters of the investigated structures (e.g., the geometry and resistivity) would influence substantially the results of a survey (Martorana et al. 2009).

Several studies have been carried out regarding the performance and efficiency of various electrode arrays configurations. Also, the merits and drawbacks of some commonly used array have been compared in order to select the most appropriate array(s) for a particular survey (Olayinka and Yaramanci 1999; Loke 2002). Some commonly used array types in resistivity studies are Wenner, Schlumberger, Wenner-Schlumberger, dipole-dipole, pole-dipole, and pole-pole (e.g., Telford et al. 1990; Reynolds 1997; Sharma 1997). It is generally recognized that Wenner and Schlumberger arrays are less sensitive to noise and have high vertical resolution whereas dipole-dipole array has lower signal-to-noise ratio but better lateral resolution (Barker 1979; Dahlin and Zhou 2004). Roy and Apparao (1971); Barker (1989) studied the depth of detection of different array types. The resolution and accuracy of the inverted data sets were investigated (Sasaki 1992; Beard and Tripp 1995; Beard et al. 1996; Candansayar and Basokur 2011; Dahlin and Zhou 2004). Also, Ward (1990) reviewed of the performance of four arrays namely dipole-dipole, pole-dipole, Schlumberger and Wenner on some geologic structures.

Park and Van (1991) emphasized the difficulty of acquiring noiseless data in the field using pole-pole arrays. Also, a qualitative evaluation of the performance of direct current resistivity for geologically complex 2D model was carried out and shown that dipole-dipole has better resolution than Wenner and Schlumberger while pole-pole array gives the poorest resolution (Seaton and Burbey 2002). Oldenburg and Li (1999) analyzed the depth of investigation of pole-pole, pole-dipole and dipole-dipole arrays. Imaging resolution using Wenner data density was examined by (Dahlin and Loke 1998) while the reliability of two-dimensional inversion of apparent resistivity data was carried out using Wenner array (Olayinka and Yaramanci 2000).

To adequately image the subsurface using electrical resistivity methods, pseudo-section resistivity datasets must be inverted using available inversion techniques (Loke and Barker 1996a) in order to produce a model which is as close as possible to the true model. With the inversion process, there are still uncertainties in the reliability of the final image. This is because inversion of electrical resistivity data is a non-linear problem and solutions are obtained using linear methods (Tarantola and Valette 1982; Tarantola 1987; Loke and Barker 1996b). In theory, the combined inversion of geophysical datasets coming from different arrays obtained at the same location would allow the comparison of the relative advantages of every array thus producing better results (Athanasiou et al. 2007). However, the use of 2D combined inversion algorithms on several datasets leads to dominance of some arrays datasets over others (Athanasiou et al. 2007). It has been established that to gain the apparent advantages of electrode arrays, it becomes imperative to combine inversion results of these datasets with a view to improving the model resolution and better reconstructing the resistivity of the model (Candansayar 2008).

In spite of the advancement made in modeling of geophysical data sets, only a few have attempted to combine multiple data sets. One of such few attempts is the use of joint inversion of geophysical data sets on the basis of gradient-based relationships that images the subsurface structures based on parallel parameter changes (Chen et al. 2006; Day-Lewis et al. 2005; Gallardo and Meju 2003,2004; Linde et al. 2006; 2006). Also, the use of a visualization approach based on the employment of computer software that automatically registers the coordinates of the data sets using their colors and opacities has been studied (Daniels et al. 2003). Haber and Oldenburg (1997) developed a structural approach to jointly inverted different data sets on the assumption that the physical properties of the models tend to change at the same location or point.

In remote sensing, merging of higher spatial resolution data with lower resolution one was used to significantly sharpen the spatial detail in an image and enhance the discrimination of features. The merging of datasets or images has provided an increased interpretation capabilities since datasets with different characteristics are combined leading to more reliable results (Genderen and Pohl 1994; Yocky 1996). Another area where image processing technique is useful is in the monitoring of urban growth. To achieve this, Intensity Hue Saturation (HIS) transformation data was combined with SPOT data (Carper et al. 1990 and Melack et al. 1994).

In this paper, a set of two-dimensional (2-D) inverse resistivity models obtained from three electrode array configurations namely: the dipole-dipole (Dpd), Schlumberger (Sch), and Wenner (Wen) for some synthetic models were combined using Algorithm Librarian of image processing technique with a view to assessing the reliability of reproducing 2-D resistivity models.

### Theoretical background

The steps involved in the stimulations and the subsequent assessment of the inversions processes are given as follows:

### Forward modeling

The models used are subdivided into a number of rectangular blocks arranged in such a way as to reflect the changes in resistivity distribution and to allow for reliable estimation of the potential difference variations across the region (Martorana et al. 2009). The calculation of the apparent resistivity data used in constructing pseudo-sections was carried out using RES2DMOD software developed by Loke and Barker 1996a. The simulations were performed with 40 electrodes at spacing of 1 m for three electrode array configurations.

### Inversion modeling

Inversion attempts to reconstruct subsurface features from a given set of geophysical measurements, and to do so in a manner that the model response fits the observations according to some measure of error. The necessary conditions for inversion of any geophysical data sets are a fast forward algorithm required for calculating theoretical data from input model parameters, and technique for calculating derivatives of the data with respect to the model parameters known as the Jacobian or derivative matrix. Among the numerous methods available in the computation of Jacobian matrix is the use of the finite differences to approximate the partial derivatives. This includes the computation of the model responses for each value of model parameters. The drawback of this approach is the long time required for computation. Narayan et al. (1994) proposed the use of perturbation analysis and reciprocity method which results in algebraic equation in the construction of the Jacobian matrix. The detailed explanations of this work are found in Narayan et al. 1994 and Olayinka and Yaramanci 2000).

## Methodology

The following gives the description of the steps involved in this study (i) creation of synthetic geological models, (ii) calculation of the 2-D forward responses and inversion process, (iii) import of inversion results datasets in ASCII files format into PCI Geomatica, (iv) image pre-processing and image registration, (v) creation of new (hybrid) combined images, (vi) image overlaying, and (vii) accuracy assessments.

### Modeling process

The procedure for the synthetic modeling study was as follows: four synthetic geologic models were created on the basis of the assumed resistivity distribution of the subsurface which was used to calculate the apparent resistivities by employing three electrode configurations namely, the Dipole-dipole(Dpd), Schlumberger (Sch), and Wenner (Wen). The synthetic forward responses then served as input to inversion process. The numerical modeling was carried out using RES2DMOD a 2-D resistivity/IP forward modeling program. The inversion process tried to find a model for the subsurface whose response agreed with the measured data subject to certain conditions. The 2-D inversion modeling was carried out using RES2DINV, a commercially widely available inversion program (Loke and Barker 1996a). The inversion routine used was based on the Guass-Newton smoothness constrained least- squares method for *L*_2_-norm optimization and the smoothness constrained, iteratively reweighted least-squares method for L_1_-norm optimization (Loke et al. 2003). The L_1_- norm optimization method allows models with sharper variations in resistivity and it is a better optimization choice when geological discontinuities are expected (Seaton and Burbey 2002; Loke et al. 2003). The L_1_-norm optimization method was used in this study. In all the models, forty electrodes with an electrode spacing of 1 m were used. A summary of the parameters used during the inversion processes with RES2DINV software is given in Table 1.

**Table 1:** **Summary of parameters used during 2-D resistivity inversions (modified after Martorana et al.** 2009 **)**

Initial damping factor	0.25
Minimum damping factor	0.015
Convergence limit	1.00
Minimum change in absolute error	
Number of iterations	5
Jacobian matrix is recalculated for first two iterations	
Increase of damping factor with depth	1.0500
Robust data inversion constrain is used with cut off factor	0.05
Robust model inversion constrain is used with cut-off factor	0.005
Extended model is used	
Effect of side blocks is not reduced	
Normal mesh is used	
Finite difference method is used	
Number of nodes between adjacent electrodes	4
Logarithm of apparent resistivity used	
Reference resistivity used is the average of minimum and maximum values	
Gauss - Newton optimization method	

### Simulations of synthetic data

To investigate the imaging capabilities of the three array configurations at resolving geometries and reconstructing resistivities of some geologic structures which are useful in exploration, archaeological, and environmental geophysics, and four synthetic models representing some geological or environmental situations were used. The model mesh used has 13 layers. A half space homogeneous resistivity (background) of 10 Ωm was used as the starting model for all the models. In synthetic modeling, a common way of evaluating a model is to consider the model misfit between the true model and inversion results. A reasonably small misfit is a necessary condition for accepting the model as a close approximation to subsurface geology (Olayinka and Yaramanci 2000). Since the purpose of inversion of resistivity data was to recover the true resistivity in the subsurface, a common way of evaluating the model was to look at the misfit between the true model and inversion results. This can be achieved by using either the standard least-square constraint which attempts to minimize the square of the difference between the observed and calculated apparent resistivity values, or a robust constraint which is less sensitive to very noisy data points (Loke et al. 2003). The four geologic synthetic models used in this study are discussed as follows.

#### One block model

**Figure 1:**
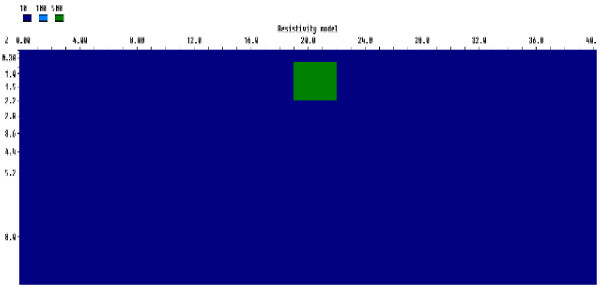
**Generic rectangular prism for a resistive block model.**

The first model simulated was a resistive block prism embedded in a low resistivity half space homogenous medium. The resistivity of the block was 500 Ωm and the surrounding background was 10 Ωm. The block prism was positioned between the 19th and 22nd electrodes. The dimension of the block was 3 m by 1.7 m and was buried below a depth of 0.5 m. The generic model used for the numerical simulation is presented in Figure 1.

#### Two blocks model

**Figure 2:**
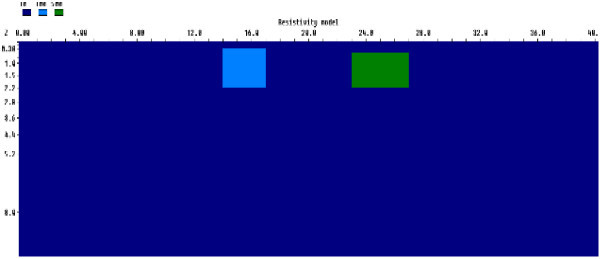
**Generic rectangular prism for two resistive blocks model.**

This model consists of two resistive blocks with resistivity values of 100 Ωm and 500 Ωm for the left and right blocks respectively embedded in a homogeneous background with resistivity of 10 Ωm. The left block prism was positioned between the 14th and 17th electrodes with thickness of 1.92 m. On the other hand, the right block was placed between 17th and 27th electrodes with a thickness of 1.7 m (Figure 2).

#### Three blocks model

**Figure 3:**
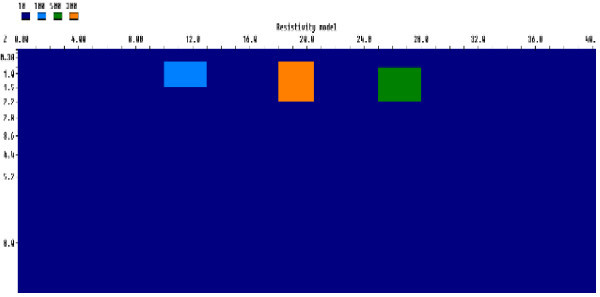
**Generic rectangular prism for three resistive blocks model.**

This model simulated a three blocks of different shapes and resistivities as shown in Figure 3. The resistivities of the blocks prisms were 100 Ωm, 300 Ωm and 500 Ωm for the left, middle and right blocks respectively. The blocks were embedded in a homogenous half space conductive medium with resistivity of 10 Ωm. The forward response of the model was used to generate the synthetic resistivity dataset (Figure 3).

#### A resistive dyke

**Figure 4:**
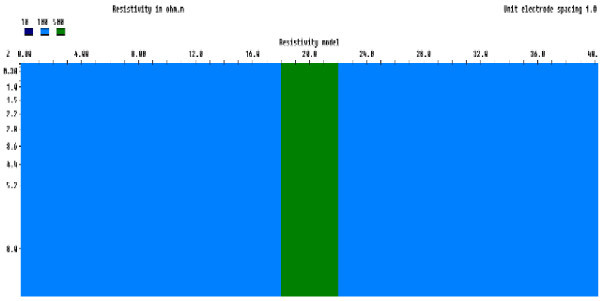
**Generic rectangular prism for a resistive dyke model.**

This model which consists of an intrusive vertical dyke of resistivity 500 Ωm across a homogeneous medium with resistivity of 100 Ωm was simulated. The geologic model and physical properties were drawn from the geophysical model proposed by (Adepelumi et al. 2006). The synthetic forward responses generated serve as input into the inversion process (Figure 4).

### Application of image processing technique

The importance of this study lies in the use of an image processing package, PCI Geomatica 10.3 version (i.e. licensed to Universiti Sains Malaysia, Penang, Malaysia) software commonly employed in remote sensing for image processing of data sets. More specifically in this effort, our goal is to explore the possibility of using the Algorithm Librarian of the software to combine the 2-D inverse resistivity models obtained from different electrode array configurations. To this end, the 2-D inverse resistivity models data sets saved in an ASCII format or text files i.e. containing information (e.g., the electrode positions (x), pseudo-depths (y), and resistivity values (ρ)) of each model were exported through the utility/transfer/translate submenu of the software and were used as input images.

As part of the preprocessing stage, the data sets were geo-referenced to the same coordinate system. In order to make further data analysis possible, the inverse models datasets were re-sampled to have a similar resolution of 0.01 m pixels along both X and Y coordinate using the natural neighbor interpolation (NNINT) scheme. For this reason, the same pixel can be compared from any combination of models’ images and statistical algebra can be performed. Image registration is one of the important stages in the image processing procedure. It is a process of matching two or more images so that corresponding points in the images correspond to the same physical region of the scene or object being imaged (Djamdji et al. 1993; Fonseca and Manjunath 1996). To register the images, we established a coordinate transformation that related the pixel coordinates (row and column) of dipole-dipole inverse models with that of Wenner and Schlumberger. The choice of dipole-dipole inverse model was informed by the fact that it was the shallowest (i.e., having the smallest pseudo-depths) but it has the largest number of data points. Finally, clipping of inverse model data sets from the other two arrays was carried out to ensure that proper overlap of the inverse models.

The datasets of the Wenner, Schlumberger and dipole-dipole models were overlaid using the utility/translate/transfer layers window of Geomatica Focus software. The general approach used for combining the data sets in this study is illustrated as follows:

Given that *ρ*_*dpd*_(*x*_*i*_, *y*_*i*_), *ρ*_*wen*_(*x*_*i*_, *y*_*i*_) *and ρ*_*sch*_(*x*_*i*_, *y*_*i*_) represent the reconstructed resistivity of the data sets from the three electrode arrays with coordinates(*x*_*i*_, *y*_*i*_). The minimum, maximum, median and average of the resistivities of the combined images are obtained as:1234

where *i* = 1…*N* is the number of data points . With these approaches, four (4) new images (combined) that might be containing features of the three individual images were obtained. The images were produced by transferring the output files to raster format. Importantly, to ensure geometrical correspondence with the same region, all raster layers have a data type with same width and height. Also, bitmap layers representing the blocks as targets, and background were created for models. Bitmap layers were used as pseudo-images and delineating masks area for the images. For each image, error images were obtained as the difference between the true model and the seven images. The absolute error (AE) which is the absolute value of resistivity between the true block and the predicted block for each point in the images was estimated. For all the data points, the mean absolute error (MAE) which is the average of the absolute error for each of the seven images was also estimated. Similarly, the mean absolute percentage error (MAPE) was computed. The EASI Modeling submenu window of the software was used to implement this task.

The final stage of the image processing procedure employed in this study was an evaluation of the accuracy of the images produced for the geologic models. This was carried out through the Histogram window of the software. To achieve this, four criteria were used namely: the mean absolute error, mean absolute percentage error, the displacement of the targets (i.e. blocks of the models to be reconstructed) as well as the mean resistivity values of the blocks. Both the MAE and MAPE were estimated using the equations below:56

where *ρ*_*i*_ is the actual resistivity of the true block and  is the predicted resistivity of the block and N is the total number of data points.

Ideally, for a good model resolution both the location and orientation of the reconstructed blocks should match exactly the position of the true blocks. However, due to models’ inadequacy the positions of the reconstructed blocks in some cases do not coincidence with the actual block’s position. As part of this study, the extent to which the recovered blocks matches the true position of the block was by measuring the vertical displacement of the centre of the reconstructed block(s) relative to the centre of the true block(s) as it was apparent that some of the reconstructed block(s) seemed to be either shallower or deeper than the position of the true block(s).

## Results and discussion

**Figure 5:**
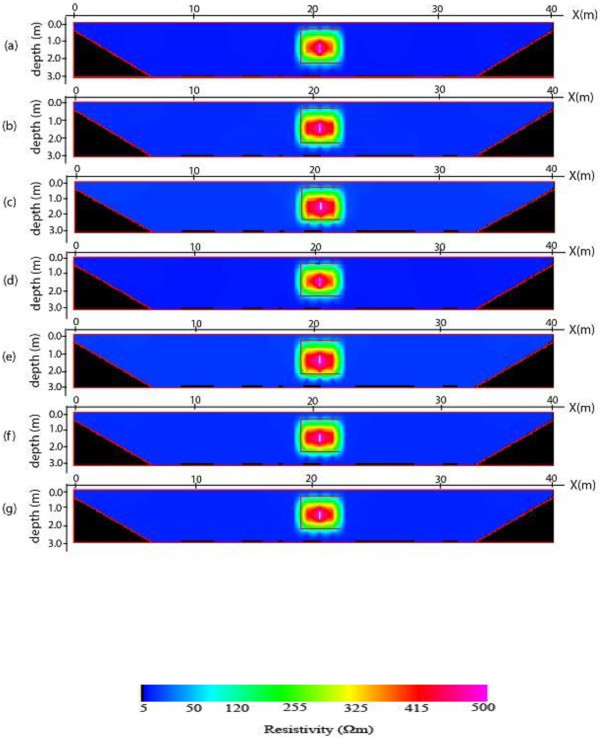
**2-D inverse resistivity model of one block images for individual (a) Dpd (b) Sch (c) Wen and for combined (d) Max (e) Min (f) Med (g) Avg of the electrode array data sets.**

**Figure 6:**
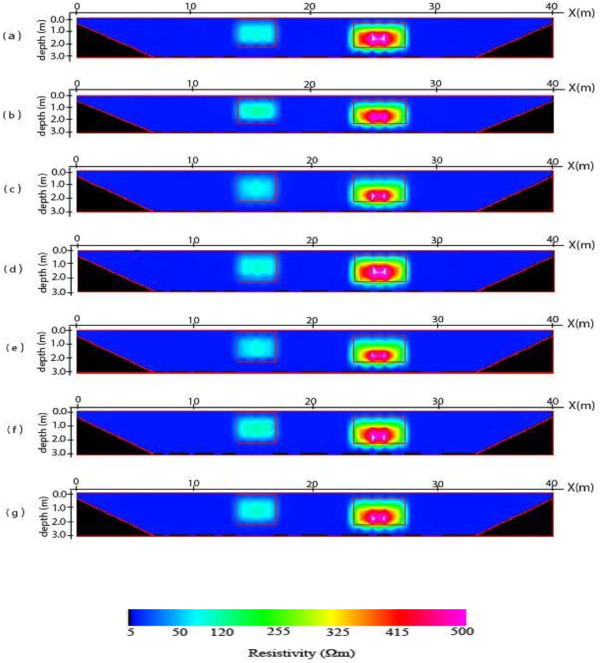
**2-D inverse resistivity model of two block images for individual (a) Dpd (b) Sch (c) Wen and for combined (d) Max (e) Min (f) Med (g) Avg of the electrode array data sets.**

**Figure 7:**
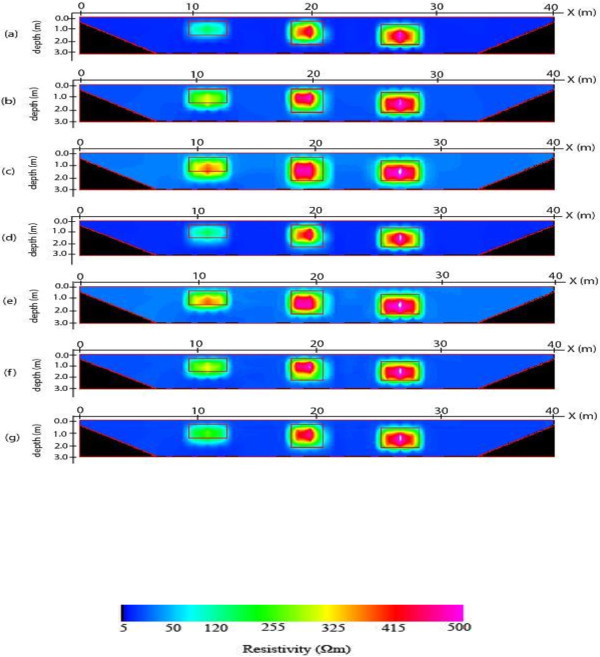
**2-D inverse resistivity model of three block images for individual (a) Dpd (b) Sch (c) Wen and for combined (d) Max (e) Min (f) Med (g) Avg of the electrode array data sets.**

**Figure 8:**
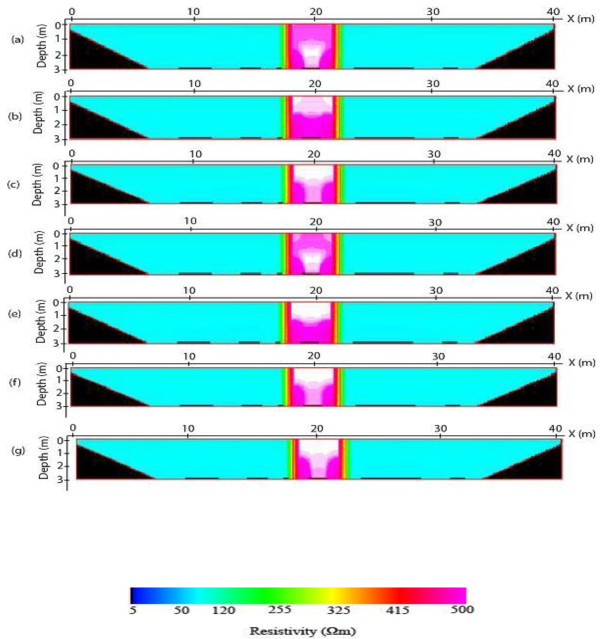
**2-D inverse resistivity model of a dyke images for individual (a) Dpd (b) Sch (c) Wen and for combined (d) Max (e) Min (f) Med (g) Avg methods of electrode array data sets.**

The 2-D inverse resistivity model images obtained after processing with the image processing package are shown in Figures 5, 6, 7 and 8. The reconstructed resistivity values of blocks and their vertical displacements relative to the true models positions are presented in Tables 2 and 3 respectively. Also, the estimated errors using the mean absolute error (MAE) and mean absolute percentage error (MAPE) as criteria for assessing the accuracy of the models’ images are summarized as Tables 4 and 5 respectively. The importance of using the PCI Geomatica for image processing lies in its ability not only to indicate or display resistivity variations across the models but also to estimate errors in attempting to reproduce the block(s), and background for all the models. This is a step ahead of most of the commercially available 2-D inversion software as more information about the model parameters can be obtained using the image processing package.

**Table 2:** **Summary of reconstructed resistivity for models**

Model name	True Resistivity (Ωm)		Mean resistivity of block (Ωm)					
		Dpd	Sch	Wen	Max	Min	Med	Avg
	Block (500)	302.2	287.9	237.9	302.9	237.8	282.8	297.5
One block	Background (10)	10.2	11.3	10.9	10.1	10.8	11.1	11.3
	Block 1 (100)	99.7	91.4	78.2	100.4	73.8	89.7	89.2
Two Blocks	Block 2 (500)	383.4	301.4	252.5	387.9	251.1	301.8	313.3
	Background (10)	16.5	15.3	15.9	17.5	14.6	15.4	15.7
	Block 1 (100)	70.3	59.3	49.9	70.4	49.4	58.5	59.0
Three Blocks	Block 2 (300)	257.1	238.5	237.9	257.2	236.5	226.3	231.0
	Block 3 (500)	398.4	340.2	321.7	399.2	321.4	339.7	341.9
	Background (10)	14.2	13.0	13.2	15.6	11.7	12.8	13.2
Dyke	Block (500)	470.6	469.1	470.1	471.7	465.9	467.9	467.9
	Background (100)	102.9	102.7	102.3	102.1	102.2	102.3	102.4

**Table 3:** **Vertical displacement of models**

Model name	Model parameter		Displacement of blocks (m)					
		Dpd	Wsc	Wen	Max	Min	Med	Avg
One block	Block	0.125	0.165	0.196	0.122	0.195	0.166	0.158
	Block 1	0.065	0.075	0.105	0.063	0.125	0.069	0.068
Two Blocks	Block 2	0.295	0.306	0.385	0.292	0.425	0.315	0.303
	Block 1	0.125	0.265	0.375	0.121	0.129	0.254	0.235
Three Blocks	Block 2	0.008	0.015	0.185	0.005	0.195	0.035	0.045
	Block 3	0.179	0.235	0.244	0.174	0.216	0.234	0.226

**Table 4:** **Summary of Mean Absolute Error (MAE) for models**

Model name	Model parameter		Mean Absolute Error (MAE)					
		Dpd	Sch	Wen	Max	Min	Med	Avg
	Block	204.7	317.1	362.0	204.0	362.1	317.1	292.5
One block	Background	3.2	2.4	2.5	3.0	2.4	2.7	2.8
	Block 1	31.9	38.9	32.9	32.0	33.8	36.8	33.1
Two Blocks	Block 2	198.5	216.4	259.2	197.1	259.0	216.3	210.0
	Background	7.7	6.5	7.4	8.4	6.3	7.0	7.3
	Block 1	31.7	41.1	50.5	31.5	50.5	41.4	40.9
Three Blocks	Block 2	143.5	216.9	232.5	142.7	236.5	213.6	197.4
	Block 3	302.1	396.2	425.8	301.7	325.8	396.3	375.0
	Background	3.5	3.9	4.3	3.1	3.4	4.2	4.5
Dyke	Block	26.3	31.2	28.6	26.0	31.5	29.6	29.5
	Background	4.2	4.5	4.8	4.1	5.0	4.7	4.9

**Table 5:** **Summary of Mean Absolute Percentage Error (MAPE) for models**

Model type	Model parameter		Mean Absolute Percentage Error (Ωm)					
		Dpd	Sch	Wen	Max	Min	Med	Avg
	Coverage	26.8	28.5	27.6	26.5	27.3	29.2	29.9
One block	Block	40.5	63.0	72.0	40.1	70.0	63.0	58.1
	Background	31.8	34.2	34.8	30.9	34.5	37.1	38.5
	Block 1	32.9	38.9	32.0	32.0	33.8	36.8	33.0
Two Blocks	Block 2	35.3	42.9	51.4	35.1	51.5	42.9	41.6
	Background	73.4	74.8	75.9	72.2	76.2	76.8	77.2
	Block 1	31.7	41.1	50.5	31.5	50.5	41.4	40.9
Three Blocks	Block 2	47.5	72.0	77.2	47.3	78.5	70.9	65.6
	Block 3	60.8	78.9	84.7	60.0	84.7	78.9	74.6
	Background	32.7	38.6	33.5	31.1	33.7	41.5	45.0
Dyke	Block	5.4	5.9	5.3	5.2	6.0	5.5	5.5
	Background	4.2	4.5	4.8	4.1	4.9	4.7	4.7

The images of a resistive block model are shown in Figure 5a-g. The images show that the geometries of the block are well resolved. The block of the dipole-dipole image nearly matches the true block than Schlumberger and Wenner (Figure 5a-c). The reconstructed resistivity of the model images ranges from 237.89 to 302.85 Ω*m* indicating that the resistivity of the true block (500 Ω*m*) is underestimated (Table 2). However, the reconstructed resistivity of the block resulting from maximum image is closer to the true model followed by dipole-dipole while the Wenner image gives the least representation. Although, the depth of burial of the block is resolved, the reconstructed block appears deeper than the actual block’s position. The vertical displacement of the block from the actual position of the block for all the seven images is presented in Table 3. The Table shows that the block of maximum image is least displaced at about 0.122 m from the centre of the true model followed by the block in image of dipole-dipole with displacement of 0.125 m while the block in the inverse model has the biggest displacement of 0.196 m from the center of the true block. As presented in Tables 4 and 5, the maximum image gives the smallest errors in trying to reproduce the block and background of the model. This is closely followed by dipole-dipole image for the individual arrays. Thus, taking into account the quality of the image, the mean resistivity, the estimated errors and the position of the block relative to the true block position, the image of maximum approach provides more reliable and detailed information about the block model than the rest of the images. As a result, it is the most appropriate approach for imaging the resistive block models followed by dipole-dipole image.

The images of the two blocks model are shown in Figure 6a-g. The geometries of the blocks are fairly resolved in all the images. It is observed that the maximum and dipole-dipole images almost replicate the true geometries of the blocks. Also, the reconstructed resistivity values for the blocks show that the best representation of blocks is obtained from the maximum image followed by dipole-dipole image, while Wenner image gives the lowest resistivity values of the blocks (Table 2). The vertical displacement of the reproduced blocks relatively to the true model blocks is in good agreement with both the mean values of the block resistivities and estimated errors regarding the three inverse model images (Table 3). The blocks in dipole-dipole image give the least displacements than Schlumberger and Wenner images. For the combined images, the blocks of the maximum images give the least displaced with values of 0.063 m for the left block and 0.262 m for the right block. Overall, the blocks of the maximum images have the least displacement from the true blocks. Imaging of the two blocks model could best be achieved by maximum approach than using any of the individual inverse models. This is also evident from the values of the estimated errors of the seven images which are presented in Tables 4 and 5 for the MAE and MAPE respectively. It is noted that the estimated errors of both maximum and dipole-dipole images give least errors, with the error indexes for maximum images least than the rest of the images. These values indicate the closeness of maximum image to the true model.

The three blocks model images are shown in Figure 7a-g. It is worth mentioning that the geometries of the blocks are fairly resolved in all the images. On the basis of fitness to model, the reconstructed blocks from images of dipole-dipole inverse models and combined maximum values nearly fit the position of the true blocks indicated by solid lines. For all the images, the reconstructed resistivity values of the three blocks are underestimated. This is a reflection of the effect of the background resistivity on the blocks. In spite of this underestimation, the mean resistivity values of the three blocks for dipole-dipole inverse model and combined maximum values image are closer to the true resistivity of the blocks while the images of Wenner inverse models and the combined minimum values of the three inverse models are not very close to the true resistivity of the three blocks (Table 2). Furthermore, the vertical displacement of the blocks from the center of the true blocks is provided in Table 3. It is observed that the blocks appear deeper than the true blocks for this model. The displacement of the blocks from the true blocks is least for image of maximum followed by dipole-dipole while Wenner and minimum images are considerably displaced from the true blocks. The estimated errors both MAE and MAPE as measure of accuracy of the images are presented in Tables 4 and 5. For the three blocks, both MAE and MAPE give the least error indexes for maximum image followed by image of dipole-dipole inverse model in attempting to reconstruct the three blocks of the model. Consequently, the maximum image gives the best representation of the three blocks model followed by dipole-dipole image.

The images of the vertical resistive dyke model are shown in Figure 8a-g. The vertical boundaries of the intrusive block (target) are well replicated in all the images. The dipole-dipole imagine is characterized by relatively small anomaly (Figure 6a) followed by Wenner inverse model image while it is more noticed in Schlumberger inverse model image. For the combined images, maximum image also shows the presence of a small anomaly which might not cause any significant change in the model parameter compared to the other images where the extent of the anomaly is considerable to be ignored especially for median and average images. Also, the reconstructed resistivity values of the dyke model also show a similar trend to the estimated errors (Table 2). The range of the resistivity is from 465.97 to 471.72 Ω*m*. Although, the block resistivity is underestimated, the results show that the reconstructed resistivity values of the block in maximum image is closer to the true resistivity of the models. Unlike the afore-mentioned models, there is no noticeable difference in the position of the reconstructed block (dyke) and true dyke, so an estimation of the displacement for these models is insignificant. Suggestively, the image of the combined maximum values and dipole-dipole inverse model are most appropriate for imaging the intrusive resistive dyke model. An evaluation of the accuracy of the images, on the basis of the estimated errors is presented in Tables 3 and 4 as MAE and MAPE respectively. The maximum and dipole-dipole images again give least estimated errors for this model. Hence, the choice of any particular array or their combination depends heavily on geologic structure to be investigated.

## Conclusion

We have presented an approach that combines the images of the 2-D inverse models resulting from three different electrode arrays (i.e. the Wenner, Schlumberger and dipole-dipole) which produced four new images (maximum, minimum, median and average) using the algorithm librarian of an image processing software. Overall, seven images were produced and analyzed for the different geologic models. The abilities of the images of the three inverse models and their combined images at resolving the geometries and reconstructing the resistivity values of the blocks have been compared and evaluated. An evaluation of the accuracy of the images was carried out through the mean absolute error, mean absolute percentage error and the apparent displacement of the blocks. The reconstructed resistivity of the blocks show that the images produced by the combined approach of using the resistivity values of maximum in Equation 1, replicate as close as possible the true blocks of the models. Hence, are most appropriate for imaging the features of the geologic models as more reliable and detailed information about the targets could be obtained.
